# Funktionelle Ergebnisse nach transmandibulärer Resektion und primärer Radiochemotherapie bei fortgeschrittenen Kopf-Hals-Plattenepithelkarzinomen

**DOI:** 10.1007/s00106-020-00930-0

**Published:** 2020-09-14

**Authors:** A. Knopf, N. Mansour, B. Hofauer, F. Johnson, H. Bier, F. Wakonig, S. Teutsch

**Affiliations:** 1 Klinik und Poliklinik für Hals‑, Nasen- und Ohrenheilkunde, Klinikum rechts der Isar der Technischen Universität München, Killianstraße 5, 79106 München, Deutschland; 2 Klinik für Hals‑, Nasen- und Ohrenheilkunde, Universitätsklinikum Freiburg, Medizinische Fakultät, Freiburg, Deutschland

**Keywords:** Kopf-Hals-Neoplasien, Qualität der medizinischen Versorgung, Qualitätsverbesserung, Chirurgische Onkologie, Lebensqualität, Head neck neoplasms, Quality of health care, Quality improvement, Surgical oncology, Quality of life

## Abstract

**Ziel der Arbeit:**

In dieser Studie wurden die funktionellen Ergebnisse nach transmandibulärer Resektion und Rekonstruktion mittels mikrovaskulär anastomosiertem Radialistransplantat (TMR+Tx) gegenüber einer primären Radiochemotherapie (pRCT) bei fortgeschrittenen orophayngealen Plattenepithelkarzinomen (OPSCC) verglichen.

**Methoden:**

Es erfolgte ein Vergleich zwischen 50 OPSCC-Patienten mit TMR+Tx und 50 OPSCC-Patienten mit pRCT. Die Wasserschluckzeit war als primärer Endpunkt definiert. Der Saxon-Test, die maxillomandibuläre Distanz, der Mallampati-Score, der Quotient aus Körpergröße zu Gewicht, eine nasale Penetration, das Vorhandensein einer Ernährungssonde/Tracheostomie sowie 4 validierte Fragebögen (Visuelle Analogskala zur Mundtrockenheit, Sicca VAS; MD Anderson Dysphagia Inventory, MDADI; Voice Handicap Index, VHI; European Organization for Research and Treatment of Cancer Quality of Life Questionnaire – Head and Neck Cancer – 35 Items, QLQ-H&N35) dienten als sonstige Endpunkte zur Beurteilung der Funktion und Lebensqualität.

**Ergebnisse:**

Die Gesamtkohorte an operierten Patienten zeigte eine erhöhte Rate an nasaler Penetration (*p* < 0,0001), was mit einer verlängerten Wasserschluckzeit verbunden war (*p* < 0,05). Durch eine modifizierte Rekonstruktion des Weichgaumens wurde die nasale Penetration in der chirurgisch therapierten Gruppe im Vergleich zur klassischen Rekonstruktion signifikant reduziert (*p* = 0,0001). Patienten mit pRCT litten signifikant stärker an einer Xerostomie (Saxon-Test) als Patienten nach TMR+Tx und Adjuvanz (*p* = 0,001). In den Fragebögen zur Funktionalität und Lebensqualität zeigte sich kein Unterschied zwischen den Gruppen.

**Schussfolgerung:**

Die TMR+Tx mit modifizierter Rekonstruktion und adjuvante Therapie zeigte gegenüber der pRCT vergleichbare funktionelle Ergebnisse.

## Abstract (en)

**Objective:**

The aim of this study was to assess the functional outcome after transmandibular resection and reconstruction via a radial forearm flap (TMR+RFF) compared to primary chemoradiotherapy (pCRT) in advanced oropharyngeal squamous cell carcinoma (OPSCC).

**Methods:**

The study compared 50 OPSCC patients treated with TMR+RFF to 50 OPSCC patients receiving pCRT. The time taken to swallow water served as the primary endpoint. The Saxon test, maxillomandibular distance, Mallampati score, ratio of height to weight, nasal penetration, tracheostomy/gastral tube requirement, and four validated questionnaires (visual analog scale for xerostomia, Sicca VAS; MD Anderson Dysphagia Inventory, MDADI; Voice Handicap Index, VHI; European Organization for Research and Treatment of Cancer Quality of Life Questionnaire, Head and Neck Cancer, 35 items, QLQ-H&N35) were applied to access functionality and quality of life.

**Results:**

Patients after TMR+RFF showed an increased rate of nasal penetration (*p* < 0.0001), which was associated with a longer water swallowing time (*p* < 0.05). A modified reconstruction of the soft palate significantly decreased the rate of nasal penetration in comparison to classical reconstruction (*p* = 0.0001). Patients with pCRT suffered significantly more xerostomia (Saxon test) than patients with TMR+RFF and adjuvant therapy. None of the subjective questionnaires revealed significant differences between the groups.

**Conclusion:**

TMR+RFF with modified reconstruction and adjuvant treatment and pCRT showed comparable functional outcomes.

Mit etwa 600.000 weltweiten Neudiagnosen pro Jahr zählen Plattenepithelkarzinome der Kopf-Hals-Region („head and neck squamous cell carcinomas“, HNSCC) zu den 6 häufigsten Malignomen [1]. Während die Inzidenz von HNSCC im Bereich vieler Lokalisationen in den letzten Jahrzehnten abgenommen hat, ist die Inzidenz von oropharyngealen Plattenepithelkarzinomen („oropharyngeal squamous cell carcinomas“, OPSCC) gestiegen, was sich wesentlich auf einen relativen und absoluten Anstieg von OPSCC, induziert durch humane Papillomviren (HPV), zurückführen lässt [2]. Aufgrund des hohen Anteils nodalpositiver Fälle wird die Mehrzahl der OPSCC mit einem fortgeschrittenen Tumorstatus diagnostiziert, was ein multimodales Therapiekonzept zur Folge hat [3]. Die Therapieoptionen umfassen chirurgische Konzepte, in aller Regel unterstützt von einer adjuvanten Radio(chemo)therapie, R(C)T, bis hin zu einer primären RCT, pRCT [4]. Bei beiden therapeutischen Konzepten ist die Balance zwischen einer ausreichenden Radikalität und dem Erhalt der funktionellen Integrität zu wahren bzw. wiederherzustellen, gleichbedeutend mit dem Erhalt der Schluck‑, Atem- und Sprechfähigkeit. Wenngleich sich einige Studien in der Rekrutierung befinden, gibt es gegenwärtig nur wenig Evidenz durch prospektive randomisierte Studien, die die Chirurgie und Adjuvanz gegenüber einer pRCT vergleichen. Exemplarisch sei an dieser Stelle eine Studie von Iyer et al. aufgeführt, die 1990 initiiert wurde und einen prospektiven randomisierten Ansatz wählte, jedoch aufgrund unzureichender Rekrutierung abgebrochen werden musste [5]. Mithin ist an dieser Stelle darauf hinzuweisen, dass die pRCT exzellente Therapieerfolge in der Untergruppe HPV-induzierter OPSCC erzielt [2]. Es bleibt jedoch kritisch anzumerken, dass es berechtige Sorge am Nachweisverfahren der HPV-induzierten Karzinogenese gibt, die sich zum einen darauf begründet, dass in der Klassifikation lediglich die Positivität des p16-Surrogatparameters gefordert wird und ferner auf Transskriptebene nur unzureichend die Inkorporierung viralen Erbguts in das Wirtsgenom differenziert wird [6, 7]. Somit bleibt zum heutigen Zeitpunkt festzuhalten, dass es keine prospektive randomisierte Studie gibt, die eine onkologische Überlegenheit der pRCT oder der Chirurgie mit Adjuvanz belegte. Gleichwohl lässt sich trefflich über funktionelle Beeinträchtigungen oder Toxizität nach den unterschiedlichen Behandlungsmodalitäten von OPSCC diskutieren. Traditionsgemäß wird der Erhalt der Funktion in der Therapie von HNSCC der pRCT zugesprochen. Insbesondere die Kollegen der Strahlentherapie berichten jedoch über eine schwere „Spättoxizität“ in den ersten 3 Monaten nach pRCT und mahnen zu einer kritischen Betrachtung [8–11]. Ein veritabler Anteil der Patienten (43 %) litt an therapiebedingten Komplikationen mit Schwierigkeiten beim Schlucken, Atmen und Sprechen [11]. In diesen Studien ist es üblich, die Spättoxizität 3 Monate nach Therapie zu beurteilen. In jüngster Vergangenheit wurde auf einen linearen Anstieg lebensbedrohlicher Spättoxizitäten Jahre nach erfolgreich abgeschlossener pRCT hingewiesen, die eine Arteriosklerose der A. carotis communis und interna, das „carotidblowout syndrome“ und das Auftreten strahleninduzierter Malignome beinhalten. Aufgrund der Biogenität der prognostisch günstigen HPV-positiven OPSCC treten Spättoxizitäten hier signifikant häufiger auf, was die Idee des Funktionserhalts nachhaltig konterkariert und die Idee des Funktionserhalts an die Chirurgie readressiert [12, 13].

Das Ziel, RCT-induzierte Spättoxizität zu minimieren, erzwingt zwangläufig die suffiziente und klare R0-Resektion, da diese, weit häufiger als der Kapseldurchbruch im Lymphknoten, zu einer adjuvanten Therapieeskalation führt, die eine energiereiche additive Strahlentherapie und in aller Regel die Hinzunahme platinhaltiger Chemotherapie zur Folge hat [14].

Die TMR ermöglicht eine exzellente Exposition des Tumors bei lokal fortgeschrittenen OPSCC, und die pharyngeale Rekonstruktion gibt die Möglichkeit, die Funktion zu erhalten oder weitestgehend wiederherzustellen. Einige Studien zeigten allerdings eine erhebliche Patientenmorbidität [15, 16].

In dieser prospektiven Studie wurden die funktionellen Ergebnisse bei Patienten mit OPSCC, die entweder eine pRCT (primäre Radiochemotherapie) oder eine TMR+Tx (transmandibuläre Resektion und Rekonstruktion mittels mikrovaskulär anastomosiertem Radialistransplantat) und eine adjuvante Therapie erhalten haben, miteinander verglichen, um die Frage zu klären, ob ein derart ausgedehntes chirurgisches Konzept einen sinnvollen Beitrag dazu leisten kann, bei vergleichbaren funktionellen Ergebnissen gegenüber der pRCT die Langzeittoxizität zu minimieren.

## Material und Methoden

### Patientenselektion

In diese Studie wurden prospektiv 100 Patienten mit OPSCC konsekutiv eingeschlossen, die in der Klinik für Hals‑, Nasen- und Ohrenheilkunde zwischen Januar 2008 und Dezember 2016 behandelt wurden. Alle Patienten litten an einem Erstmalignom im Kopf-Hals-Bereich ohne bereits stattgehabte Therapie und waren zum Zeitpunkt der Untersuchung rezidivfrei. Von den Patienten wurden 50 operiert und erhielten eine TMR+Tx. Nach ausführlicher Beratung entschieden sich 50 weitere Patienten, die zwar potenziell mittels TMR+Tx operabel waren, für eine pRCT. Im Fall einer adjuvanten RCT wurde diese als perkutane intensitätsmodulierte Strahlentherapie nach CT-gestützter Bestrahlungsplanung bis 50 Gy (Einzeldosis, ED: 2 Gy, 5 Fraktionen/Woche) der regionalen Lymphabflusswege und des Primärtumors sowie Boost der Primärtumorregion und befallener Lymphknoten auf 64 Gy durchgeführt. Bei einer R1-Resektion oder einem Kapseldurchbruch im Lymphknoten wurden 40 mg/m^2^ Cisplatin verabreicht. Eine primäre Radiotherapie wurde perkutan, intensitätsmoduliert, nach CT-gestützter Bestrahlungsplanung bis 50 Gy (ED 2 Gy, 5 Fraktionen/Woche) der regionalen Lymphabflusswege und des Primärtumors sowie mit zusätzlichem Boost der Primärtumorregion und befallener Lymphknoten auf 70,4 Gy (ED 2,2 Gy) simultan zu einer möglichen cisplatinhaltigen Chemotherapie (40 mg/m^2^ 1 ×/Woche) durchgeführt. Bei sehr ausgedehnten Bestrahlungsvolumina wurde die Strahlentherapie auf eine kumulative Gesamtdosis von 66 Gy beschränkt. Patienten mit primär inoperablem OPSCC wurden ausgeschlossen. Die Tumorproben wurden durch mindestens 2 erfahrene Pathologen beurteilt. Dysplasien, Carcinoma in situ und andere histologische Subtypen wurden ausgeschlossen. An klinischen Parametern wurden Alter, Geschlecht, Noxenexposition, TNM-Status entsprechend der 7. Auflage, das Grading und die Behandlungsmodalitäten erhoben.

Die Studie wurden von der Ethikkommission geprüft und genehmigt (Nr. 389/16S).

### Repräsentative Kohorte

Zur Betrachtung des Gesamtüberlebens wurde eine repräsentative Kohorte an Patienten gewählt, die zwischen 2001 und 2011 aufgrund eines Oropharynxkarzinoms therapiert wurden. Der Nachbeobachtungszeitraum betrug mehr als 60 Monate. Überschneidungen mit der hier funktionell untersuchten Kohorte lagen nicht vor.

Ein‑/Ausschlusskriterien wurden wie folgt definiert:

- Keine vorangegangene Kopf-Hals-Tumor-Therapie
- Resezierbarer Primärtumor und lokale Metastasen
- T > 1 N1–3 M0
- Ausschluss von Patienten mit abgebrochener Therapie

### Objektive funktionelle Tests

Um den Einfluss der Tumortherapie auf die Funktionalität zu evaluieren, mussten die Patienten einige objektive Tests durchlaufen. Die Wassertrinkzeit diente als primärer Endpunkt und wurde bei jedem Patienten ermittelt. Dabei sollte jeder Patient 100 ml Wasser so schnell, wie er es vermochte, trinken, ohne eine Aspiration oder nasale Penetration zu provozieren. Der Test, der gut etabliert ist, hat eine hohe Validität in der Identifikation von posttherapeutischer Aspiration und in der quantitativen Untersuchung von Schluckproblemen bei Patienten mit Kopf-Hals-Malignomen über die Zeit [17]. Eine Wassertrinkzeit von <3,23 ml/s wurde als pathologisch definiert.

Zusätzlich wurde auch der Grad der nasalen Penetration während des Trinkens mittels einer funktionellen endoskopischen Schluckuntersuchung (FEES) beurteilt. Die Mundtrockenheit wurde mittels Saxon-Test evaluiert [18]. Hierbei wurde die Differenz des Gewichts eines Schwämmchens vor und nach 2 min Kauen im Mund des Patienten ermittelt. Zudem wurden die maxillomandibuläre Distanz (Kieferöffnung) von Gingiva zu Gingiva und der Mallampati-Score, die Notwendigkeit einer Ernährungssonde (perkutane endoskopische Gastrostomie-Sonde, PEG-Sonde) oder Tracheostomie und der Quotienten zwischen Größe und Gewicht als Marker des Ernährungszustands ermittelt.

### Subjektive funktionelle Tests

Um den Einfluss der verminderten Funktionalität auf die tägliche Routine und die Lebensqualität beurteilen zu können, sollte jeder Patient 4 validierte Fragebögen zu Stimmproblemen, zum Essen, zur Mundtrockenheit und zum generellen Gesundheitszustand beantworten.

Der QLQ-H&N35-Fragebogen (European Organization for Research and Treatment of Cancer Quality of Life Questionnaire – Head and Neck Cancer – 35 Items) beinhaltet 35 Fragen und evaluiert typische Symptome von Patienten nach HNSCC-Behandlung. Er beinhaltet Fragen nach Schmerzen, Schluckproblemen, Gefühlsstörungen, Sprechproblemen, Problemen beim Essen in der Öffentlichkeit, Problemen damit, soziale Kontakte zu knüpfen, verminderter Sexualität, Zahnproblemen, Problemen mit der Mundöffnung, Problemen mit Mundtrockenheit und zähem Schleim, Husten, Krankheitsgefühl, Gebrauch von Schmerzmitteln und Nahrungsergänzungsmitteln, Gewichtsverlust und -zunahme. Jede Kategorie wurde einzeln von 0–100 (0 = sehr geringe Symptome) bewertet. Der QLQ-H&N35 wurde den entsprechenden Empfehlungen nach beurteilt [19].

Der Sicca-VAS-Fragebogen beinhaltet 8 Fragen, die v. a. die Mundtrockenheit beurteilen. Die Symptomschwere wurde auf einer visuellen Analogskala von 0–100 angegeben. Es wurden Mittelwerte für jeden Patienten errechnet [20]. Der Test beinhaltete folgende Fragen/Aufgaben:

- F1. Beurteilen Sie Ihre Schwierigkeit beim Sprechen in Bezug auf Mundtrockenheit.
- F2. Beurteilen Sie Ihre Schwierigkeiten beim Kauen in Bezug auf Mundtrockenheit.
- F3. Beurteilen Sie Ihre Schwierigkeiten beim Schlucken fester Speisen in Bezug auf Mundtrockenheit.
- F4. Beurteilen Sie die Häufigkeit von Schlafproblemen in Bezug auf Mundtrockenheit.
- F5. Beurteilen Sie Ihre Mund- oder Halstrockenheit beim Essen von Speisen.
- F6. Beurteilen Sie Ihre Mund- oder Halstrockenheit, wenn Sie nicht essen.
- F7. Beurteilen Sie die Häufigkeit des Trinkens, um das Schlucken von Essen zu erleichtern.
- F8. Beurteilen Sie die Häufigkeit des Trinkens von Flüssigkeiten zur Befeuchtung der Mundschleimhaut, wenn Sie nicht essen.

Der MD Anderson Dysphagia Inventory (MDADI) besteht aus 20 Fragen, die Probleme mit dem Schlucken abfragen. Er enthält 4 Kernbereiche: den globalen, emotionalen, funktionellen, und den physischen Bereich. Jeder Kernbereich wird einzeln von 0–100 beurteilt (0 = sehr geringfunktionierend) [21].

Der Voice Handicap Index (VHI) ist ein validierter Test, um Sprechprobleme zu beurteilen. Er enthält 30 Fragen, die tägliche Probleme in sozialer Interaktion beschreiben und von 0 (keine Probleme) bis 120 (sehr ausgeprägte Sprechprobleme) beurteilt werden [22].

### Modifizierte pharyngeale Rekonstruktion

Die hier untersuchten Oropharynxkarzinome erzwangen eine kraniale Absetzung, die den Weichgaumen medial des Befunds durchgreifend durchtrennte. Über die Flügelgaumengrube und dem Spatium parapharyngeum wurde die tiefe Absetzung definiert und nach kaudal im Bereich der Oropharynxsseitenwand abgesetzt. Über diese kaudale Absetzung wurde die Schnittführung nach rostral in Richtung Zungengrund überführt und im Bereich des Sulcus glossoalveolaris die Schnittführung zur Absetzung des Gaumens über 360° geschlossen.

Im Bereich der kraniodorsalen Absetzung (Oropharynx-Seitenwand zu nasopharyngealem Weichgaumen) resultierte ein großer Defekt in Richtung Nasenrachen.

**Figure 1:**
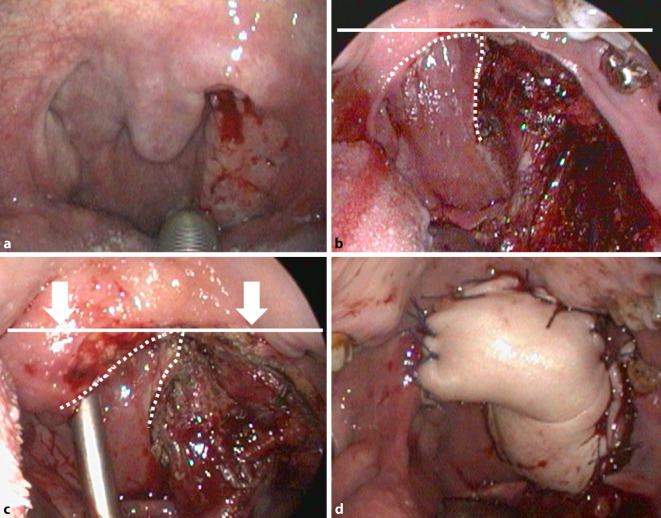

Eine Gruppe von Patienten erhielt die klassische Rekonstruktion mittels Radialistransplantat, das zirkulär entlang der pharyngealen Schleimhautabsetzung eingenäht wurde. Die zweite Gruppe der Patienten erhielt eine modifizierte Rekonstruktion mittels Radialistransplantat, um die nasale Penetration zu minimieren. Hierbei wurde gezielt die kraniodorsale Absetzung nach kaudal verlagert, indem die kraniale Absetzung der Oropharynx-Seitenwand gegen das nasopharyngeale Blatt des residuellen Weichgaumens abgenäht wurde. Durch dieses Manöver wurde nicht nur der Scheitelpunkt der am weitesten kranial gelegenen Schleimhaut kaudalisiert, sondern auch die Choane verkleinert (Abb. 1). Anschließend erfolgte auch hier das Einnähen des Radialistransplantats an die Absetzungsränder. In beiden Techniken wurde durch Dopplung des Radialistransplantats dieses überkorrigiert, um den Schrumpfungseffekten durch Narbenzug und adjuvanter Strahlentherapie entgegenzuwirken.

### Statistik

Unterschiede zwischen den Gruppen wurden mittels χ^2^-Test und Fisher-Exakt-Test für kategoriale und der ungepaarte und gepaarte T‑Test für numerische Werte durchgeführt. Variablen mit prognostischem oder einflussnehmendem Potenzial auf die Endpunkte wurden mittels vorwärtsgerichteter Cox-Regression beurteilt. *p*-Werte <0,05 wurden als statistisch signifikant betrachtet. Statistische Analysen wurden mittels SPSS durchgeführt (Fa. SPSS Inc., Chicago/IL, USA).

## Ergebnisse

### Gesamtüberleben

**Figure 2:**
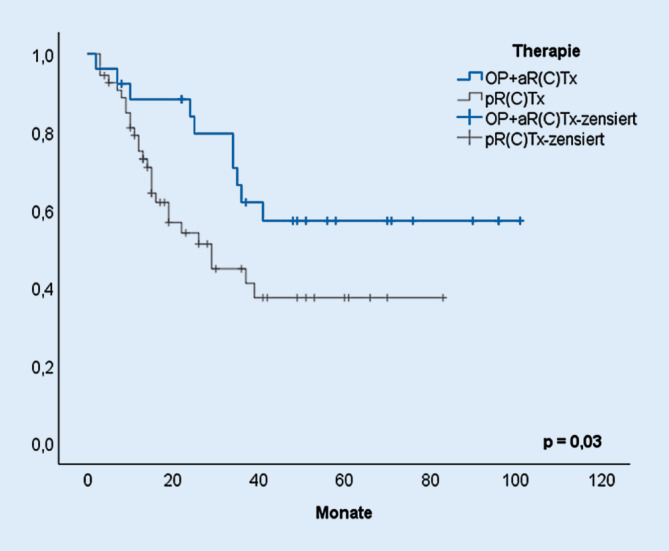

Aus der Gesamtkohorte an Patienten, die zwischen 2001 und 2011 aufgrund eines HNSCC therapiert wurden, konnten 80 Patienten in die Auswertung einbezogen werden, die die Einschlusskriterien erfüllten. Bei 26 Patienten wurde eine transmandibuläre Resektion und Defektdeckung via mikrovaskulär anastomosiertem Radialistransplantat durchgeführt, komplettiert durch eine Neck-Dissection und adjuvante R(C)T. Weitere 54 Patienten mit potenziell operablem OPSCC durchliefen vollständig eine primäre R(C)T. Im Hinblick auf das mittlere Gesamtüberleben zeigte sich mit 69 Monaten (Konfidenzintervall, KI: 53; 84) eine signifikante Überlegenheit der Chirurgie mit Adjuvanz gegenüber einer primär konservativen Therapie, für die ein mittleres Überleben von 42 Monaten (KI: 32; 52) beobachtet wurde (*p* = 0,03; Abb. 2).

### Epidemiologie der Kohorte

In diese Studie wurden 100 Patienten mit fortgeschrittenem OPSCC eingeschlossen. Prinzipiell waren alle Patienten via TMR+Tx operabel. Alle Patienten erhielten nach Abschluss des prätherapeutischen Stagings eine Beratung durch den Kopf-Hals-Chirurgen und den Strahlenonkologen. Schlussendlich wurden 50 Patienten mit TMR+Tx sowie anschließender adjuvanter Therapie 50 Patienten mit pRCT gegenübergestellt. Die mittlere Zeit zwischen Tumortherapie und Analyse der funktionellen Ergebnisse betrug 29 und 36 Monate ohne Unterschiede zwischen den Gruppen (*p* = 0,26; Tab. 1). In den Gruppen zeigte sich kein Unterschied in Alter und Geschlecht. Das mittlere Alter war 59 Jahre mit einem Geschlechtsverhältnis zugunsten des männlichen Geschlechts von 76–77 % (Tab. 1). Die Mehrzahl der Patienten hatte eine Vorgeschichte von Nikotinabusus mit durchschnittlich 28 Packyears. Es waren signifikant mehr Raucher in der Gruppe der konservativ therapierten Patienten (28 % aktive Raucher, 58 % Nikotinkonsum; *p* = 0,02; Tab. 2).

**Table 1:** 

	Op. + R(C)T	Primäre R(C)T	*p*-Wert
*Patientenanzahl (n)*	50	50	–
*Zeitraum Therapie bis Nachuntersuchung (Monate)*	–	–	0,26
MW ± SD (Median)	29 ± 29 (22)	36 ± 38 (20)	–
*Alter (Jahre)*	–	–	0,51
MW ± SD (Median)	60 ± 10 (59)	61 ± 9 (%)	–
*Geschlecht, n (%)*	–	–	0,91
Männlich: weiblich	40 (80)/10 (20)	38 (76)/12 (24)	–
*cT-Status, n (%)*	–	–	0,70
T1	0	1 (2)	–
T2	17 (34)	14 (28)	–
T3	21 (42)	19 (38)	–
T4	12 (24)	15 (30)	–
*pT-Status, n (%)*	–	–	0,03
T1	0	–	–
T2	34 (68)	–	–
T3	12 (24)	–	–
T4	4 (8)	–	–
*Tumorlokalisation*	–	–	0,67
Tonsille	35 (70)	33 (66)	–
Weichgaumen	24 (48)	17 (34)	–
Mundhöhle	7 (14)	5 (10)	–
Zungengrund	12 (24)	15 (30)	–
*cN-Status, n (%)*	–	–	0,83
N0	0	3 (6)	–
N1	1 (2)	7 (14)	–
N2	49 (98)	40 (80)	–
*pN-Status, n (%)*	–	–	0,01
N0	9 (18)	–	–
N1	11 (22)	–	–
N2	30 (60)	–	–
*M‑Status, n (%)*	–	–	0,31
M0	50 (100)	50 (100)	–
*Grading, n (%)*	–	–	0,16
G1	0	2 (4)	–
G2	25 (50)	25 (50)	–
G3 G4	25 (50) 0	17 (34) 6 (12)	–
*R‑Status, n (%)*	45 (90)	–	–
R0	5 (10)	–	–
R1	–	–	–
*Adjuvante Therapie, n (%)*	–	–	–
RT	27 (54)	–	–
RCT	23 (46)	–	–
*Primär konservative Therapie, n (%)*	–	–	–
RT	–	16 (32)	–
RCT	–	34 (68)	–

*R(C)T *Radio(chemo)therapie,* MW* Mittelwert, *SD* Standardabweichung

**Table 2:** 

	Op. + R(C)T	Primäre R(C)T	*p*-Wert
*Alkoholkonsum (ml/Tag)*	690	630	0,69
Kein Konsum, *n* (%)	17 (27)	14 (28)	0,71
Zustand nach, *n* (%)	15 (30)	16 (32)	–
Aktiv, *n* (%)	20 (40)	20 (40)	–
*Art des Alkoholkonsums*			
Bier, *n* (%)	31 (62)	29 (58)	0,59
Wein, *n* (%)	5 (10)	7 (14)	–
Spirituosen, *n* (%)	1 (2)	1 (2)	–
*Nikotinkonsum (Packyears)*	23	32	–
Kein Konsum, *n* (%)	18 (36)	7 (14)	0,02
Zustand nach, *n* (%)	25 (50)	29 (58)	–
Aktiv, *n* (%)	9 (18)	14 (28)	–

*R(C)T *Radio(chemo)therapie,* MW* Mittelwert, *SD* Standardabweichung

Auch im klinischen prätherapeutisch erhobenen TNM-Status (cTcN) zeigte sich zwischen den Gruppen kein signifikanter Unterschied. Ein cT3/4- und cN+-Status wurden bei 66 bzw. 100 % der Patienten mit chirurgischer Therapie und bei 68 bzw. 94 % der Patienten mit pRCT diagnostiziert. In der chirurgisch therapierten Gruppe zeigte sich allerdings ein signifikanter Unterschied zwischen dem klinischen und pathologischen TNM-Status (pTpN). Nach Aufarbeitung der histologischen Präparate durch mindestens 2 erfahrene Pathologen wiesen lediglich 32 und 82 % ein T3/4-Status bzw. N+-Status auf (*p* = 0,03 bzw. *p* = 0,01). Im Hinblick auf die Ausbreitung der Primärtumoren zeigte sich in beiden Untersuchungsgruppen insbesondere eine tonsilläre und velare Manifestation. Ein Befall des Zungengrunds ließ sich bei 12 % bzw. 15 % der Patienten beobachten. Aufgrund der gewählten Einschlusskriterien war insbesondere der kombinierte Befall von Tonsille und Weichgaumen zu beobachten, wohingegen ein Ausbreitungsmuster in Richtung Sulcus glossoalveolaris selten auftrat (*p* = 0,67, Tab. 1).

Keiner der Patienten wies eine Fernmetastasierung zum Zeitpunkt der Erstdiagnose auf. Nach chirurgischer Therapie erhielten 54 % der Patienten in dieser Gruppe eine (alleinige) adjuvante RT und 46 % eine adjuvante RCT. Bei 10 % der chirurgisch therapierten Patienten konnte der Tumor nicht im Gesunden (R1-Status) reseziert werden. Hierbei zeigte sich eine R1-Situation in 3 Fällen im Bereich der Flügelgaumengrube sowie in 2 Fällen im Bereich des Mundbodens mit Übertritt zum Level Ib, allesamt korrespondierend zum tiefen Absetzungsrand. In der Gruppe der konservativ therapierten Patienten erhielten 68 % eine pRCT und 32 % der Patienten eine alleinige pRT (Tab. 1).

### Operationsassoziierte Morbidität

Bei 8 Patienten (16 %), die eine TMR+Tx erhielten, traten Komplikationen auf, die eine operative oder medikamentöse Therapie erzwangen. Eine Nachblutung, die operativ behandelt werden musste, zeigte sich bei 3 Patienten. Bei einem Patienten zeigte sich eine Dehiszenz des transplantierten Radialislappens an der kranialen Oropharynxnaht sowie spenderseitig eine unzureichende Bedeckung der Unterarmflexoren durch das aufgebrachte Vollhauttransplantat. Das Repexieren des Lappens und die neuerliche, umschriebene Vollhautdeckung am Unterarm erfolgten in einer Sitzung. Eine Lungenarterienembolie war bei 4 Patienten klinisch apparent. Unter den 50 untersuchten Patienten war kein Lappenverlust zur beklagen (Tab. 3).

**Table 3:** 

	Op. + R(C)T
Nachblutung, *n* (%)	3 (6)
Lappenverlust, *n* (%)	0
Lappendehiszenz, *n* (%)	1 (2)
Revision spenderseitig Unterarm, *n* (%)	1 (2)
Revision spenderseitig Leiste, *n* (%)	0
Lungenarterienembolie	4 (8)

*R(C)T *Radio(chemo)therapie

### Objektive Tests

Die Wassertrinkzeit war der primäre Endpunkt. Es zeigte sich ein signifikant höheres Trinkvolumen pro Sekunde in der rein konservativ behandelten Gruppe verglichen mit der chirurgisch therapierten Gruppe (8,08 ml/s vs. 6,48 ml/s; *p* < 0,05; Tab. 4). Zusätzlich zeigte sich bei 66 % der chirurgisch therapierten Gruppe eine nasale Penetration in der FEES (*n* = 33), während nur 14 % (*n* = 7) in der konservativ therapierten Gruppe dieses Symptom aufwiesen (*p* < 0,0001; Tab. 4). Im Gegensatz dazu war im Saxon-Test in der konservativ therapierten Gruppe eine signifikant niedrigere Speichelproduktion als in der chirurgisch therapierten Gruppe zu beobachten (1,02 g/2 min vs. 1,82 g/2 min; *p* = 0,001; Tab. 4). Es zeigte sich kein signifikanter Unterschied zwischen den Gruppen bezüglich der Notwendigkeit einer Ernährungssonde oder einer Tracheostomie, der maxillomandibulären Distanz, dem Mallampati-Score und dem Quotienten aus Körpergröße und Gewicht (Tab. 4).

**Table 4:** 

	Op. + R(C)T	Primäre R(C)T	*p*-Wert
Quotient aus Körpergröße und Gewicht	2,53	2,60	0,43
Ernährungssonde, *n* (%)	5 (10)	10 (20)	0,14
Tracheostomie, *n* (%)	1 (4)	3 (6)	0,96
Wassertrinkzeit (ml/s)	6,48	8,08	<0,05
Nasale Penetration, *n* (%)	33 (66)	7 (14)	<0,0001
Saxon-Test (g/2 min)	1,82	1,02	0,001
Mallampati-Score	2,42	2,82	0,07
Maxillomandibuläre Distanz (mm)	50,35	53,46	0,15

*R(C)T *Radio(chemo)therapie

### Subjektive Tests

**Figure 3:**
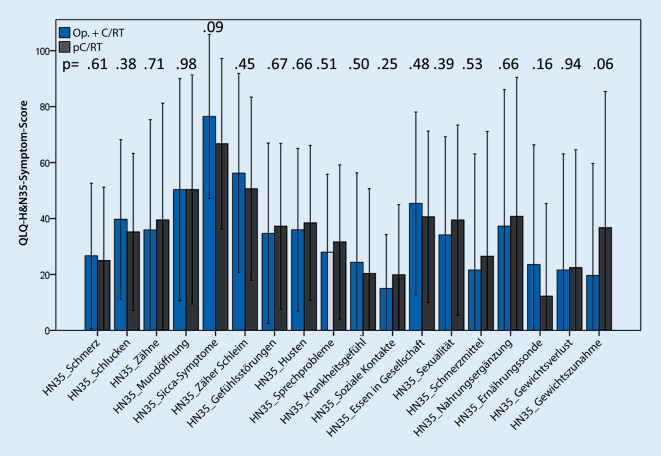

Im QLQ-H&N35-Fragebogen wurde gezeigt, dass der wichtigste Faktor für Patienten die Mundtrockenheit war. Fragen in Bezug auf Sicca-Symptome zeigten, dass die Sicca-Symptome mit Werten um etwa 78 (0 = nur sehr geringe Symptome) in beiden Gruppen für Patienten am relevantesten im Hinblick auf die Lebensqualität waren. In Abb. 3 wurden die verschiedenen Items, die in beiden Gruppen erhoben wurden, dargestellt.

**Figure 4:**
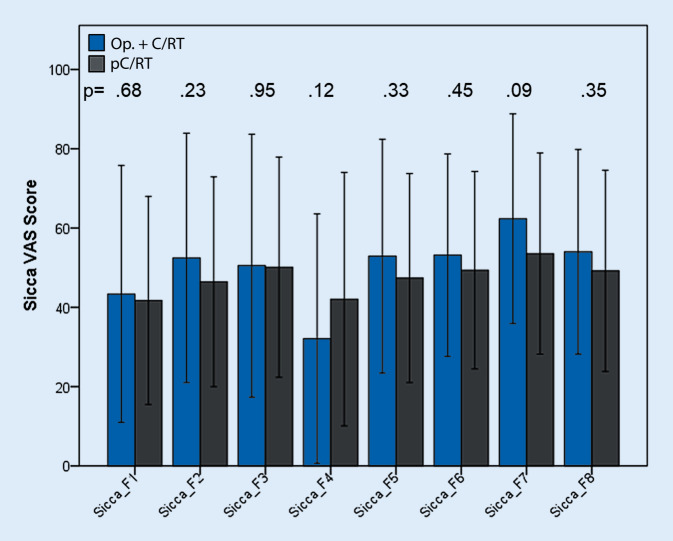

Im Sicca-VAS-Fragebogen wurde ebenfalls ein hoher Einfluss der Sicca-Symptome auf die Lebensqualität aufgedeckt. Alle Patienten litten unter Sicca-Symptomen. Dabei berichteten Patienten von einem subjektiven Beschwerdegrad von mindestens 35 (bezüglich Schlafproblemen durch Mundtrockenheit) bis hin zu einem Maximum von 65 (bezüglich Schluckproblem versursacht durch Mundtrockenheit) in beiden Gruppen. Bei diesem subjektiven Fragebogen zeigte sich im Vergleich zum Saxon-Test kein signifikanter Unterschied zwischen den beiden Gruppen (Abb. 4).

**Figure 5:**
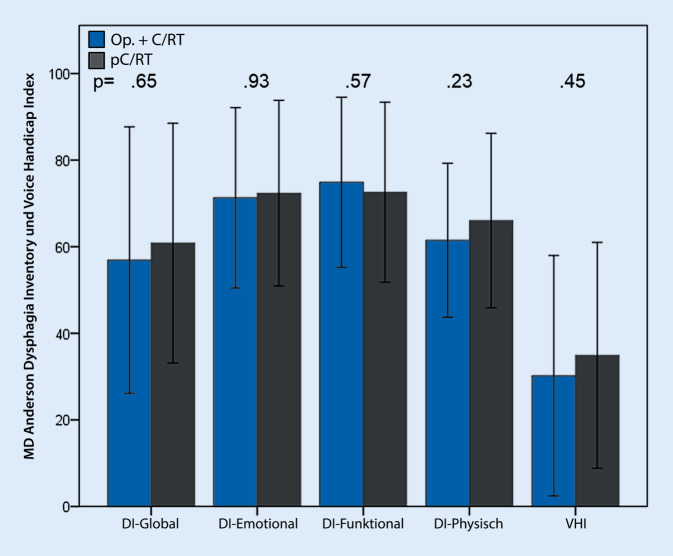

Mit dem MDADI wurde gezeigt, dass die Dysphagie ebenfalls einen wichtigen Faktor im Hinblick auf die Lebensqualität nach Therapie eines OPSCC darstellte. Die generelle Schluckqualität betrug im Mittel etwa 60/100 in beiden Gruppen. Im Hinblick auf die emotionalen, funktionellen und physischen Bereiche zeigten sich fast ähnliche hohe Werte zwischen den Gruppen (Abb. 5). Der VHI für die subjektiven Stimmprobleme zeigte nur einen moderat erhöhten Wert nach oropharyngealer Tumortherapie, mit einem durchschnittlichen Wert von 35 (120 = hohes Maß an Stimmproblemen; Abb. 5). Im Vergleich der beiden Gruppen bezüglich Schluck- und Stimmqualität in den subjektiven Tests zeigten sich keine signifikanten Unterschiede.

### Modifizierte Rekonstruktion

Die vorwärtsgerichtete Cox-Regression, die den pathologischen Saxon-Test und die nasale Penetration auf den primären Endpunkt analysierte, zeigte, dass nur die nasale Penetration mit einem 3,8-fach erhöhten Risiko für eine pathologische Wassertrinkmenge (<3,23 ml/s) einherging (*p* = 0,002). Daher wurden Subgruppenanalysen im Hinblick auf die verschiedenen rekonstruktiven Techniken durchgeführt. Dabei erhielten 25 Patienten die modifizierte pharyngeale Rekonstruktion. Es zeigte sich eine signifikante Reduktion der nasalen Penetrationsrate von 84 % (*n* = 21) in der Gruppe mit der klassischen Rekonstruktion auf 48 % in der Gruppe mit der modifizierten Rekonstruktion. Dennoch war die Anzahl an Patienten mit nasaler Penetration in der konservativ therapierten Gruppe am geringsten (*p* < 0,0001; Tab. 5).

**Table 5:** 

	Klassische Rekonstruktion	Modifizierte Rekonstruktion	Primäre R(C)T	*p*-Wert
Quotient aus Körpergröße und Gewicht	2,53	2,51	2,60	0,73
Ernährungssonde, *n* (%)	2 (8)	3 (12)	10 (20)	0,32
Tracheostomie, *n* (%)	1 (4)	0	3 (6)	0,84
Wassertrinkzeit (ml/s)	5,94	7,02	8,08	0,12
Nasale Penetration, *n* (%)	21 (84)	12 (48)	7 (14)	<0,0001
Saxon-Test (g/2 min)	1,79	1,86	1,02	0,003
Mallampati-Score	2,15	2,69	2,82	0,04
Maxillomandibuläre Distanz (mm)	50,35	50,35	53,46	0,35

*R(C)T *Radio(chemo)therapie

## Diskussion

Die Operation mit anschließender adjuvanter Therapie sowie die pRCT sind etablierte Konzepte in der Behandlung des OPSCC. Während der Funktionserhalt traditionell der pRCT zugesprochen wird, zeigten Untersuchungen eine schwerwiegende Toxizität der pRCT 3 Monate nach Therapie, insbesondere im Hinblick auf die Funktionen der Stimme, des Schluckens, des Sprechens und des Atmens. Während die Hinzunahme platinhaltiger Chemotherapie das Gesamtüberleben signifikant steigert [23], ist hiermit gleichsam eine signifikante Steigerung der Toxizität verbunden [11, 24]. Insbesondere Patienten jenseits des 70. Lebensjahrs sind häufiger von schweren Komplikationen betroffen [25]. Jüngere Studien rücken indes die Patienten mit onkologisch günstigen HPV-positiven OPSCC in den Fokus, die offensichtlich eine Prädisposition zeigen, lebensbedrohliche, gefäßassoziierte Komplikationen viele Jahre nach pRCT zu erleiden [12, 13]. Die gegenwärtig vielfach diskutierte und in Studien untersuchte Deeskalation lässt sich nicht nur in einem primär konservativen Kontext betrachten, sondern ebenso chirurgisch, da in einem adjuvanten Therapiekontext in vielen Fällen auf eine Chemotherapie verzichtet werden kann und zudem die Strahlendosis und Konturierung günstig beeinflusst werden können. Gleichsam erzwingt diese Idee die klare R0-Resektion, da die R1-Resektion, als häufigste Ursache für die adjuvante Therapieeskalation im Sinne der Hinzunahme platinhaltiger Chemotherapie und Dosiserhöhung der dann additiven Radiotherapie, der Deeskalationsstrategie à priori entgegensteht [14, 25]. Zweifelsohne ist die Option der adjuvanten Therapieeskalation eine gute und sinnvolle Option, da die R0-Situation und v. a. der Kapseldurchbruch im Lymphknoten nicht zwangsläufig zu antizipieren sind und durch eine additive RCT eine Steigerung des Gesamtüberlebens um bis zu 15 % erreicht wird [26–28]. Gleichwohl sollte diese Option nicht als erstrebenswert missverstanden, sondern vielmehr durch die geeignete Wahl des chirurgischen Zugangs auf ein Minimum beschränkt werden.

Die in dieser Studie untersuchte TMR+Tx zählt zu den umfangreichen chirurgischen Konzepten. Dieser Zugang liefert eine exzellente Tumorexposition und ermöglicht eine posterior-anteriore Dissektion nach Sicherung der A. carotis interna und damit einen kontrollierten tiefen Absetzungsrand. Zwangsläufig ist bei derart großen chirurgischen Zugängen die unmittelbare operationsassoziierte Morbidität zu beurteilen, die bei 8 Patienten (16 %) in Form von Nachblutungen, Dehiszenzen, Wundheilungsstörungen und Lungenembolien in der vorliegenden Studie beobachtet wurde. Gemessen am chirurgischen Umfang und verglichen mit der gegenwärtigen Literatur dürfte dieser Verlauf als komplikationsarm eingestuft werden, wobei es erfreulicherweise zu keinem Lappenverlust und zu einer geringen spenderseitigen Morbidität kam [15, 16, 23].

### Überlegenheit der Operation

Im Hinblick auf die onkologischen Ergebnisse zeigen die hier vorliegenden retrospektiven Daten einer repräsentativen Kohorte die Überlegenheit der TMR+Tx und Adjuvanz gegenüber der pRCT. Natürlich ist es wünschenswert, solche Studien als randomisierte Studie zu konzipieren. Die Vergangenheit hat aber gezeigt, dass Patienten insbesondere mit der zufälligen Randomisierung zu einem beträchtlichen Teil nicht zufrieden waren und deshalb gar nicht an solchen Studien partizipieren wollten oder wegen individueller und abweichender Therapiewünsche nach der Randomisierung ausgeschlossen werden mussten [5]. Der „aufgeklärte Patient“, der über alle Therapieoptionen informiert werden muss und möchte und sich dann, je nach persönlicher Situation und Faktoren, mit seinem Behandler für eine Therapie entscheidet, macht die Konzeption solcher Studien umso schwieriger.

Zentrales Moment der vorliegenden Studie war die differenzielle Betrachtung der Funktionalität nach TMR+Tx und pRCT. Den Selektionsbias versuchte die Studie dahingehend zu umgehen, dass prinzipiell für alle Patienten nach dem Staging die gleiche Operation von chirurgischer Seite indiziert wurde und die Patienten sich nach ausführlicher Beratung vom Kopf-Hals-Chirurgen und Strahlenonkologen für die jeweilige Therapie entschieden.

### Homogenität der Kohorte

Die klinische TNM-Klassifikation (cTNM-Status), die in beiden Gruppen keine signifikanten Unterschiede zeigte, unterstreicht, dass die Homogenität der Kohorte gegeben war und dass, trotz eines fortgeschrittenen Stadiums, alle Patienten prinzipiell der gleichen Therapie hätten zugeführt werden können. Dabei zeigte sich in der chirurgisch behandelten Gruppe hinsichtlich der pathologischen TN-Klassifikation (pTN-Status) ein signifikanter Unterschied zum klinischen TN-Status. Ein „Overstaging“ sowohl im T‑ als auch im N‑Status lässt sich bei Oropharynxkarzinomen regelhaft beobachten und dürfte wesentlich auf die in Deutschland übliche und hochsensitive Kombination aus Diagnostik mittels Magnetresonanztomographie (MRT) und Computertomographie (CT) sowie hochauflösendem Ultraschall zurückzuführen sein. Hierdurch wird deutlich, dass der Chirurgie, und v. a. der Neck-Dissection, nicht nur ein therapeutisches Moment zugrunde liegt, sondern sie auch wesentlichen diagnostischen Erkenntnisgewinn mitbringt, der die zielgenaue Konturierung in der häufig erforderlichen Adjuvanz erst ermöglicht [29]. Gleichsam sind primär konservative Konzepte gezwungen, zur Wahrung des onkologischen Ergebnisses eine rein bildmorphologisch gestützte Therapie durchzuführen. Darüber hinaus zeigten Umstattd et al., dass eine signifikante Schrumpfungstendenz direkt nach der Resektion der Tumoren entstand. Auch nach der Formalinfixierung kam es tendenziell zur Schrumpfung der Resektionsränder, wenngleich dieses Ergebnis nicht signifikant war [30]. Dies würde für eine Überinterpretation der Tumorgröße durch Gewebeeigenschaften und eine bestehende Gewebespannung im Situs sprechen. Kordac et al. wiesen in ihrer Studie auch nach, dass sich in 53 % der Fälle nach Operation eine unterschiedliche TNM-Klassifikation ergab [31]. Gallo et al. sahen ähnliche Ergebnisse bei Patienten mit Larynxkarzinomen bezüglich des Lymphknotenbefalls und zeigten in histologischen Präparaten eine gewisse Anzahl an nichtbefallenen Lymphknoten, die klinisch fälschlicherweise als N+ beurteilt wurden [32]. Um ein kuratives Konzept mit bestmöglicher lokaler und lokoregionärer Kontrolle und gutem Gesamtüberleben zu erreichen, bleibt dem Strahlenonkologen bei der Planung seiner primären Radiochemotherapie somit nichts anderes übrig, als sich auf die klinisch festgelegte Ausdehnung des OPSCC zu verlassen und so möglicherweise unnötig radikal zu therapieren.

In dieser vorliegenden Studie konnte bei 54 % der chirurgisch behandelten Patienten mit fortgeschrittenem OPSCC eine konkomitante adjuvante RCT eingespart werden. Von Bedeutung ist, dass bei 46 % der Patienten eine adjuvante RCT nach der Operation empfohlen wurde. Allerdings war bei nur 5 Patienten (10 %) eine additive Therapie aufgrund einer R1-Resektion erforderlich. In der MRT können nicht alle Fälle von extrakapsulärem Wachstum in Lymphknotenmetastasen aufgedeckt werden (Sensitivität von 72 %) [33]. Deshalb muss gelegentlich, trotz einer akkuraten präoperativen Diagnostik, nach einer chirurgischen Therapie eine adjuvante RCT angeschlossen werden.

### Additive Nebenwirkungen

Insbesondere die auf die Lebensqualität potenziell additiven Nebenwirkungen einer radikalen Chirurgie in Kombination mit einer adjuvanten RCT sollten vor einer transmandibulären Resektion mit dem Patienten besprochen werden, auch wenn eine Chirurgie onkologisch angemessen erscheint. In Hinblick auf die Funktion analysierte diese Studie verschiedene subjektive und objektive Tests, um die posttherapeutische Funktion 33 Monate nach Therapie zu erheben. Insgesamt zeigt sich bei den untersuchten Patienten ein hoher Leidensdruck durch die strahlenbedingte Xerostomie und Dysphagie, was in allen Fragebögen und VAS nachvollzogen werden konnte. Lediglich der VHI-Fragebogen zeigte moderate Werte als Ausdruck von nur mäßigen Sprechproblemen nach den verschiedenen Therapien eines OPSCC. Zusammenfassend zeigte keines der subjektiven Items der 4 Fragebögen einen signifikanten Unterschied zwischen den Gruppen. Dies legt die Vermutung nahe, dass trotz des großen chirurgischen Eingriffs die subjektive Lebensqualität aller Patienten hauptsächlich durch die R(C)T verursacht war, ungeachtet einer adjuvant oder primär definitiven Konzeption. Andere Studiengruppen konnten ebenfalls zeigen, dass bei fortgeschrittenen OPSCC die ausgedehnten chirurgischen und rekonstruktiven Maßnahmen keine signifikanten Auswirkungen auf die Funktion oder die Lebensqualität hatten [34, 35]. In den objektiven Tests zeigte sich, dass die quälende Mundtrockenheit sich in der Gruppe der primär konservativ therapierten Patienten im Saxon-Test objektivieren ließ und die hohen Strahlendosen auf die Speicheldrüsen reflektiert. Im Gegensatz dazu zeigte sich in der chirurgisch behandelten Gruppe eine deutlich verminderte Wassertrinkzeit. In den weiteren Regressionsanalysen war die in der FEES nachgewiesene nasale Penetration in der Gruppe von Patienten mit TMR+Tx mit einem 3,8-fach erhöhten Risiko einer reduzierten Wassertrinkzeit assoziiert. In bereits veröffentlichten Studien gibt es verschiedene Erklärungsansätze für den unzureichenden Schluss des Nasopharynx nach Chirurgie, wie etwa die geminderte Transplantatsensibilität oder die reduzierte Elevation des weichen Gaumens. Aber der meistgenannte Ansatz war die späte Nebenwirkung der adjuvanten Strahlentherapie. Dies hatte die Autoren dazu veranlasst, die Rekonstruktionstechniken zu überdenken und eine Verkleinerung des nasopharyngealen Übergangs durchzuführen. Da der höchste Absetzungspunkt der Schleimhaut den finalen Scheitelpunkt des Radialistransplantats Jahre nach Rekonstruktion zu determinieren scheint, wurden aktiv die Schleimhautkanten des dorsalen Blatts des Weichgaumens mit der Oropharynxhinterwand vernäht, hiermit die Schleimhautabsetzung kaudalisiert und der nasopharyngeale Übergang verkleinert. Ebenso wurde der antizipierten Transplantatvolumenreduktion durch eine Überkorrektur des Lappens entgegengewirkt. Durch diese Technik zeigte sich ein signifikanter Abfall der nasalen Penetration und in der Post-hoc-Analyse eine vergleichbare Wassertrinkzeit wie in der Gruppe der konservativ behandelten Patienten. Neben den allgemeinen Nebenwirkungen einer RCT war die nasale Penetration vernachlässigbar für die Lebensqualität. Es gab in keinem der subjektiven Fragebögen einen Hinweis darauf, dass dieses Symptom einen Effekt auf die Lebensqualität der Patienten hatte. Barata et al. fanden nasalen Reflux und Penetration sowie phonatorische Einschränkungen bei Patienten, bei denen eine Malignomoperation am Weichgaumen durchgeführt worden war. Auch hier zeigte die subjektive Wahrnehmung der funktionellen Einschränkung einen nur geringen Einfluss auf die Lebensqualität [36].

## Fazit für die Praxis

- Der Chirurgie der Primärtumorregion und der lokalen Lymphknoten kommt sowohl eine therapeutisches als auch diagnostisches Moment zu und bildet so das Fundament für eine Reduktion der strahlentherapeutischen Toxizität durch eine zielgenaue Konturierung, Verzicht auf die Chemotherapie sowie Dosisreduktion in der Adjuvanz.
- Ausgedehnte chirurgische Maßnahmen (transmandibuläre Tumorresektion) tragen wesentlich zum Erreichen der R0-Resektion bei und verhindern hierdurch die adjuvante Therapieeskalation im Sinne der additiven Radiochemotherapie.
- Die transmandibuläre Tumorresektion zeigte nach 33 Monaten ein ähnliches funktionelles Ergebnis nach modifizierter Rekonstruktion mittels Radialistransplantat wie eine pRCT.
- Der mögliche Langzeit-Benefit einer reduzierten RCT-Dosis in der Gruppe operierter Patienten kann erst nach einem längeren Beobachtungszeitraum beurteilt werden.
